# Genome-Wide Haplotype Analysis of *Cis* Expression Quantitative Trait Loci in Monocytes

**DOI:** 10.1371/journal.pgen.1003240

**Published:** 2013-01-31

**Authors:** Sophie Garnier, Vinh Truong, Jessy Brocheton, Tanja Zeller, Maxime Rovital, Philipp S. Wild, Andreas Ziegler, The Cardiogenics Consortium, Thomas Munzel, Laurence Tiret, Stefan Blankenberg, Panos Deloukas, Jeannette Erdmann, Christian Hengstenberg, Nilesh J. Samani, Heribert Schunkert, Willem H. Ouwehand, Alison H. Goodall, François Cambien, David-Alexandre Trégouët

**Affiliations:** 1 INSERM, UMR_S 937, Pierre and Marie Curie University (UPMC, Paris 6), Paris, France; 2 ICAN Institute for Cardiometabolism and Nutrition, Pierre and Marie Curie University (UPMC, Paris 6), Paris, France; 3 Department of General and Interventional Cardiology, University Heart Center Hamburg, Hamburg, Germany; 4 Department of Medicine II, University Medical Center, Johannes Gutenberg University Mainz, Mainz, Germany; 5 Institut für Medizinische Biometrie und Statistik, Universität Lübeck, Lübeck, Germany; 6 Human Genetics, Wellcome Trust Sanger Institute, Hinxton, United Kingdom; 7 Universität zu Lübeck, Medizinische Klinik II, Lübeck, Germany; 8 Klinik und Poliklinik für Innere Medizin II, Universität Regensburg, Regensburg, Germany; 9 Department of Cardiovascular Sciences, University of Leicester, Leicester, United Kingdom; 10 National Institute for Health Research Biomedical Research Unit in Cardiovascular Disease, Glenfield Hospital, Leicester, United Kingdom; 11 Department of Haematology, University of Cambridge and National Health Service Blood and Transplant, Cambridge, United Kingdom; Georgia Institute of Technology, United States of America

## Abstract

In order to assess whether gene expression variability could be influenced by several SNPs acting in *cis*, either through additive or more complex haplotype effects, a systematic genome-wide search for *cis* haplotype expression quantitative trait loci (eQTL) was conducted in a sample of 758 individuals, part of the Cardiogenics Transcriptomic Study, for which genome-wide monocyte expression and GWAS data were available. 19,805 RNA probes were assessed for *cis* haplotypic regulation through investigation of ∼2,1×10^9^ haplotypic combinations. 2,650 probes demonstrated haplotypic p-values >10^4^-fold smaller than the best single SNP p-value. Replication of significant haplotype effects were tested for 412 probes for which SNPs (or proxies) that defined the detected haplotypes were available in the Gutenberg Health Study composed of 1,374 individuals. At the Bonferroni correction level of 1.2×10^−4^ (∼0.05/412), 193 haplotypic signals replicated. 1000G imputation was then conducted, and 105 haplotypic signals still remained more informative than imputed SNPs. In-depth analysis of these 105 *cis* eQTL revealed that at 76 loci genetic associations were compatible with additive effects of several SNPs, while for the 29 remaining regions data could be compatible with a more complex haplotypic pattern. As 24 of the 105 *cis* eQTL have previously been reported to be disease-associated loci, this work highlights the need for conducting haplotype-based and 1000G imputed *cis* eQTL analysis before commencing functional studies at disease-associated loci.

## Author Summary

In order to assess whether gene expression variability could be influenced by the presence of more than one *cis*-acting SNP, we have conducted a systematic genome-wide search for haplotypic *cis* eQTL effects in a sample of 758 individuals and replicated the findings in an independent sample of 1,374 subjects. In both studies, genome-wide monocytes expression and genotype data were available. We identified 105 genes whose monocyte expression was under the influence of multiple *cis*-acting SNPs. About 75% of the detected genetic effects were related to independent additive SNP effects and the last quarter due to more complex haplotype effects. Of note, 24 of the genes identified to be affected by multiple *cis* eSNPs have been previously reported to reside at disease-associated loci. This could suggest that such multiple locus-specific genetic effects could contribute to the susceptibility to human diseases.

## Introduction

The development of high throughput technologies has stimulated comprehensive surveys on genome-wide expression and DNA variability data for disentangling the genetic architecture of human diseases [1]–[3]. The genetics of transcript abundance has been extensively investigated through genome-wide expression studies (GWES) [4]–[9]. These studies demonstrated that, for a large fraction of genes (so-called eQTLs), expression is influenced by single nucleotide polymorphisms (SNPs) located in the vicinity of the regulated genes, generally referred to as *cis* eSNPs. The importance of *cis* eSNPs would be enhanced if they were associated at the same time with a disease, as such data would indicate that the associated gene is a candidate for the disease. Despite its limitations [2], [3], [10], [11], the integration of GWES and genome wide association studies (GWAS) data has recently received great attention [12] and several successes illustrate the merits of this approach [13]–[15].

Most *cis* eQTL studies so far were based on single SNP analyses that did not account for the multiplicity of *cis* eSNPs that are often observed at an eQTL. For example, in the Gutenberg Health Study (GHS) [9] conducted on monocyte expression, the median number of eSNPs per eQTL was eleven. One way to investigate whether associations observed at several *cis* eSNPs of the same eQTL are independent, or due to linkage disequilibrium (LD) between SNPs, is to conduct haplotype analysis, a strategy shown to be able to distinguish “true” effect from those due to LD [16], [17]. Another approach is to perform GWES conditioning on the best *cis* eSNPs identified through a first run of GWES [11]. The limitation of this strategy is that it is only able to identify *cis* eSNPs that have independent additive effects, contrary to haplotype analysis which can identify combinations of SNPs having non-additive effects or tagging a rare functional variant.

**Figure 1:**
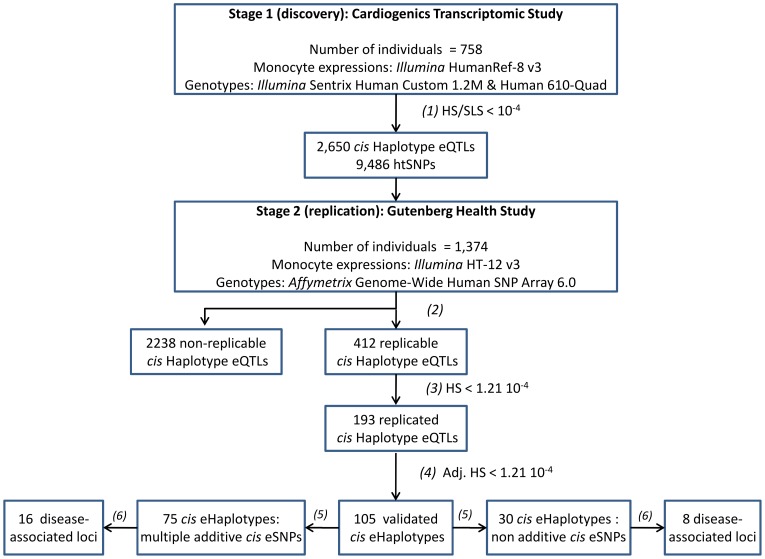
Main outlines of the research strategy for identifying *cis* haplotype effects. (1): Probes with best haplotype signal (HS) 104-fold smaller than the smallest single-locus signal (SLS) in the discovery study (CTS) were selected for replication in GHS. (2): Probes that had *cis* htSNPs available in GHS were considered as replicable. (3): Probes for which the global test of *cis* haplotypic association was significant at the Bonferroni threshold (1.21 10^−4^) in GHS and the pattern of *cis* haplotypic association was consistent between CTS and GHS (the same haplotypes with effects in the same direction) were considered as replicated. (4): *Cis* haplotypic associations were considered as validated when the haplotypic p-value was still significant at the Bonferroni threshold (1.21 10^−4^) after adjusting for the best (genotyped or imputed) SNP identified in single-locus association analysis in GHS. (5): Nested likelihood ratio tests and conditional haplotype analyses were used to check whether validated haplotype effects could be compatible with the additive effects of multiple SNPs (see Methods). (6): *cis* Haplotype eQTLs overlapping with disease-associated loci obtained from the Genome-Wide Association Studies catalog (Hindorff et al. 2009) [23]. htSNPs: haplotype tagging SNPs.

In this work, we conducted a systematic genome-wide search for haplotypic *cis*-acting effects on monocyte gene expression using data from the Cardiogenics Transcriptomic Study (CTS) [14]. A comprehensive replication of the haplotypic associations detected in CTS was then performed in the same cell type using the GHS dataset [9]. A summary of the overall research strategy adopted in this work is displayed in Figure 1.

## Results

### Research strategy

The discovery phase was conducted in CTS where monocyte gene expression profiles were assessed in 758 subjects using the *Illumina* HumanRef-8 v3 Beadchip array and genome-wide genotypes were assessed using the *Illumina* Human Custom 1.2M and Human 610 Quad Custom arrays. We analyzed 19,805 autosomal probes covering 15,428 genes. For each probe, a systematic search for *cis* haplotype effects was undertaken according to the sequential procedure described in the Methods section. Probes with strong statistical evidence for *cis* haplotype effect were selected for replication in GHS where monocyte gene expression profiles were assessed in 1,374 individuals using the *Illumina* HT-12 v3 BeadChip and genome-wide genotypes were assessed using the *Affymetrix* Genome-Wide Human SNP Array 6.0. As the CTS and GHS projects did not use the same genome-wide SNP arrays, if a SNP contributing to a *cis* haplotype effect in CTS was not genotyped in GHS, we tried to identify a proxy SNP (pairwise LD r^2^>0.80) using the SNAP software [18].

### Discovery phase

For identifying *cis* haplotype effects in CTS, we selected all SNPs located within a 200 kb distance upstream or downstream from any probe sequence (346,749 autosomal SNPs). SNPs located within a 200 kb distance of several adjacent probes were analyzed with each probe separately. The distribution of the number of SNPs per probe is shown in Figure S1, with minimum, mean and maximum values of 2, 70.9 and 287, respectively. To reduce the redundancy among SNPs due to strong LD, we selected haplotype tagging SNPs (htSNPs) within each consecutive bin of 10 adjacent SNPs (see Methods). This resulted in a subset of 181,233 htSNPs for analysis. The minimum, mean and maximum numbers of htSNPs per probe were 2, 46.9 and 187, respectively (Figure S1).

For each probe locus, we characterized all haplotypic configurations derived from the combination of 1 to 4 (not necessarily adjacent) htSNPs. These haplotypes were then tested for association with expression level of their corresponding probe, resulting in 2,097,693,183 associations explored for the 19,805 probes. This analysis was conducted using the GridHaplo software [19] on the European Grid Infrastructure EGI [20]. In order to get robust results, we focused on probes for which the best haplotypic p-value for association was at least 10^4^-fold smaller than the best single SNP p-value at the locus. This criterion was used on pragmatic grounds to select probes which would not have been picked up by a single SNP analysis and where haplotypes were likely to be more relevant than single SNP alone for explaining probe expression variability. Among the 19,805 investigated probes, 2,650 (13.4%) fulfilled this criterion. When selecting the htSNPs involved in the best haplotypic configuration at each associated locus (see Methods), the total number of associated htSNPs was 9,486. Considering more stringent thresholds of 10^6^, 10^8^, 10^10^ and 10^50^-fold difference for measuring the improvement of p-value of haplotype over single SNP analysis decreased the number of probes with *cis* haplotypic effects to 1,550 (7.8%), 1,069 (5.4%), 834 (4.2%) and 74 (0.4%), respectively.

### Replication phase

The 2,650 probes identified at the 10^4^ threshold in the discovery phase were interrogated in the GHS expression dataset. All probes were available for replication. However, among the 9,486 htSNPs characterizing the best haplotype associations in CTS, only 5,162 (54%) were directly genotyped or could be tagged by a genotyped proxy SNP in GHS. As a consequence, the replication of haplotypic signals could be assessed only in 412 of the 2,650 probes.

Replication of the haplotypic signals observed in CTS was performed using the THESIAS software [21] implementing the same statistical haplotype model as GridHaplo. We considered as replicated in GHS those probes which exhibited a significant association consistent with that observed in CTS (i.e. the same haplotypes associated with the same direction of effects on probe expression). A Bonferroni threshold correcting for the number of probes tested for replication (n = 412) was taken (p<1.21×10^−4^). At this significance level, the haplotypic effects detected in CTS were replicated in GHS for 193 of the 412 probes (46.8%).

We further investigated whether these haplotypic effects could be explained by a single SNP. For this purpose, we imputed SNPs in GHS using the MACH software [22] and taking as reference the European panel from the 1000 Genome Project. Among the 10,210,859 SNPs imputed with good quality, all those located within a 200 kb distance from a replicated probe were tested for association with their corresponding probe expression by linear regression analysis assuming additive allele effects. Conditional analyses were then conducted in which haplotype effects were adjusted for the SNP demonstrating the strongest single SNP association, i.e. the best *cis* eSNP.

For 88 of the 193 probes (45.6%), the haplotype p-value was no longer significant (p>1.21×10^−4^) after adjustment for the best *cis* eSNP, suggesting that the haplotypic signal was due to the effect of a single SNP that was missed in the discovery phase because the SNP was not genotyped in CTS. For the 105 remaining probes (54.4%), the haplotype p-value was still significant in the conditional analysis, indicating a residual effect beyond that of the best *cis* eSNP. Four different situations were encountered as outlined in Table S1.

**Figure 2:**
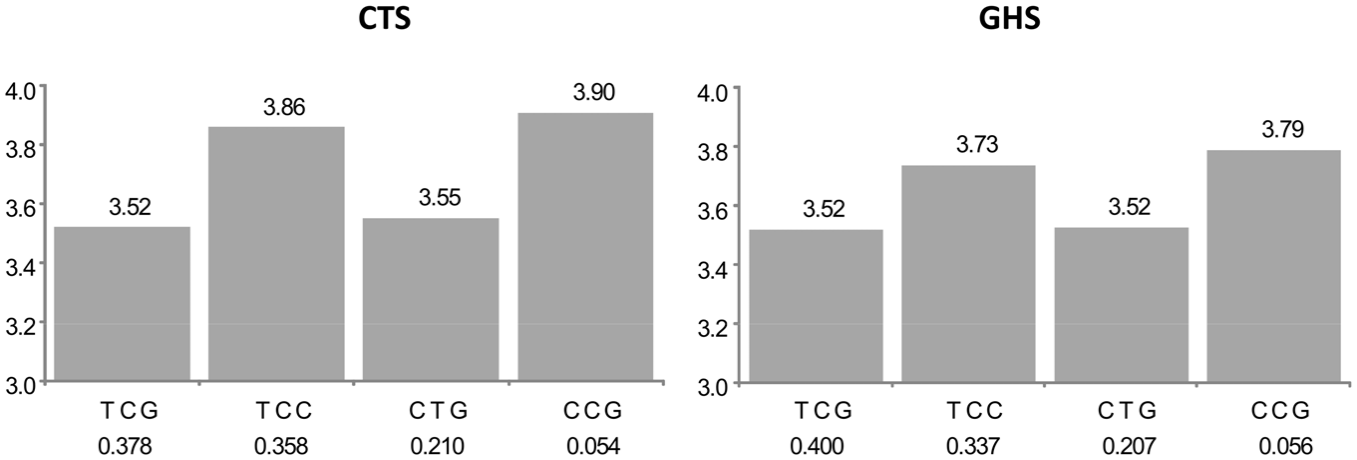
Association between ILMN_2367638 (*CAMKK2*) expression and the main haplotypes derived from rs1140886, rs1063843, and rs11065504. The left panel shows the results in the discovery cohort CTS and the right panel in the replication cohort GHS. Each bar corresponds to the expected mean of gene expression associated with one dose of the corresponding haplotype under the assumption of additive haplotype effects. According to this model, the expression level of an individual is the sum of the levels of his (her) two haplotypes. Haplotype frequencies are indicated under each haplotype label. For ease of presentation, mean expression for the most frequent haplotype in CTS was set to be the same as that observed in GHS. In CTS, the rs11065504 was substituted by its proxy rs3794207 (r^2^ = 0.96). After imputation, the best *cis* eSNP in GHS was rs11065504 whose allele C was carried by an unique haplotype, TCC, which was associated with increased *CAMKK2* expression (β = +0.338, p = 9.05 10^−156^ and β = +0.217, p = 5.69 10^−151^ in CTS and GHS, respectively) compared to the TCT haplotype. In addition, the less common CCG haplotype was associated with an even stronger increase in *CAMKK2* expression (β = +0.386, p = 5.09 10^−56^ and β = +0.269, p = 4.00 10^−53^, resp.).

For 5 of the 105 probes (group A in Table S1), the best *cis* eSNP was among the htSNPs defining the haplotypic combination associated to expression, but it was not sufficient to explain alone the observed association. Such a situation is illustrated in Figure 2 with the *CAMKK2* gene. The best haplotypic configuration is composed of 3 SNPs, rs1140886, rs1063843 and rs11065504. The best *cis* eSNP is rs11065504 whose allele C, associated to an increased expression, is carried by a single haplotype TCC. However, there is a rare haplotype CCG not carrying this allele which is associated with an even greater increase of expression. These effects are remarkably similar in CTS and GHS.

**Figure 3:**
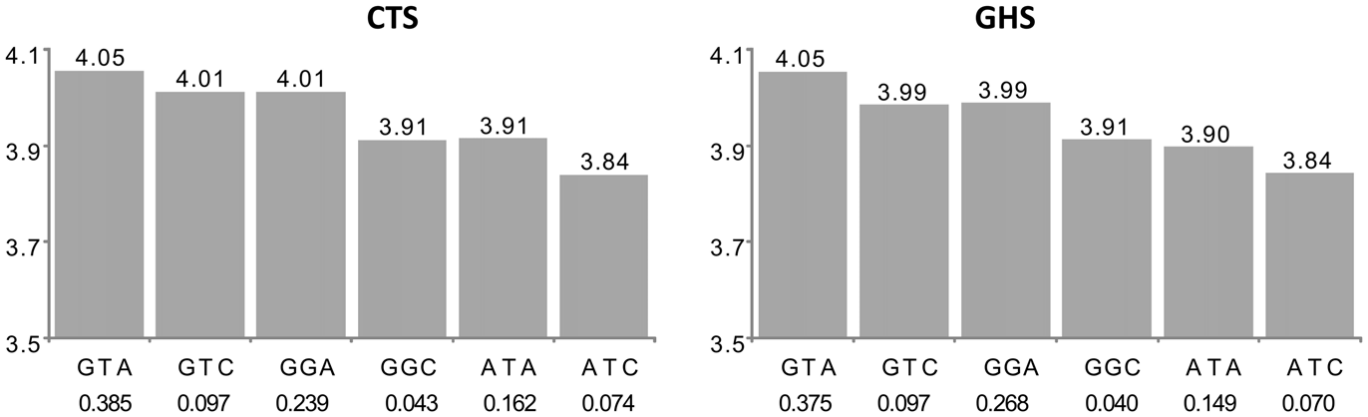
Association between ILMN_1731596 (*AP3S2*) expression and the main haplotypes derived from rs7173483, rs3803536, and rs1269077. See the first paragraph of legend in Figure 2 for explanations. In CTS, the rs7173483, rs3803536 and rs1269077 were substituted by their corresponding proxies, rs4932145 (r^2^ = 1), rs10520684 (r^2^ = 0.92) and rs1256854 (r^2^ = 0.95), respectively. After imputation, the best *cis* eSNP in GHS was rs12148357 which was not among the associated htSNPs. Its minor allele was associated with decreased *AP3S2* expression (β = −0.146; p = 1.59 10^−54^). However, in the conditional model adjusting for haplotype effects, its effect was no longer significant (β = −0.022, p = 0.420) suggesting that it was due to LD with haplotypes. The haplotypic association was compatible with the additive effects of three SNPs. The rs7173483-A allele was associated with decreased *AP3S2* expression (β = −0.147, p = 2.80 10^−18^ and β = −0.1500; p = 9.50 10^−11^ in CTS and GHS, respectively), as were the rs3803536-G allele (β = −0.052, p = 5.03 10^−4^ and β = −0.065, p = 1.75 10^−6^, resp.) and the rs1269077-C allele (β = −0.067, p = 2.93 10^−7^ and β = −0.066, p = 9.49 10^−17^, resp.).

For 19 probes (group B in Table S1), the best *cis* eSNP was not among the associated htSNPs and lost its significance in the conditional model, while the haplotypic signal was barely modified by the adjustment on the best *cis* eSNP. This suggests that the effect of the best *cis* eSNP was due to its LD with the identified haplotypes. An example is given in Figure 3 with the *AP3S2* gene.

**Figure 4:**
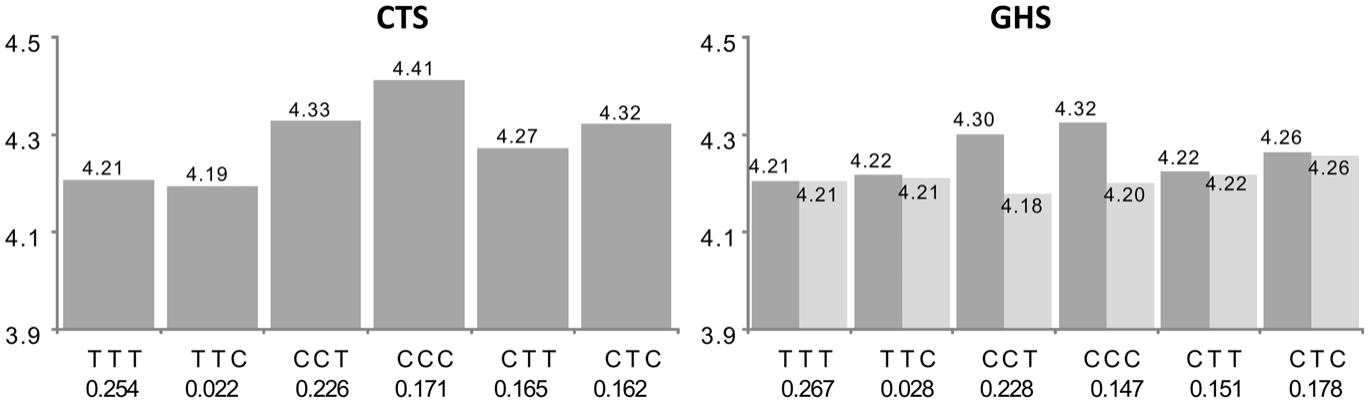
Association between ILMN_1726554 (*IREB2*) expression and the main haplotypes derived from rs1394371, rs13180, and rs950776. See the first paragraph of legend in Figure 2 for explanations. In CTS, the rs950776 was substituted by its proxy rs1948 (r^2^ = 0.96). The original haplotypic association (dark grey bars) was compatible with the effects of three common haplotypes associated with increased *IREB2* expression, CCT (β = +0.121, p = 1.75 10^−12^ and β = +0.096, p = 2.40 10^−25^ in CTS and GHS, respectively), CCC (β = +0.205, p = 2.69 10^−29^ and β = +0.118, p = 1.10 10^−30^, resp.) and CTC (β = +0.115, p = 7.87 10^−10^ and β = +0.059, p = 5.31 10^−10^, resp.). After adjusting for the best imputed *cis* eSNP rs12592111 in GHS (light grey), the effect of the CCT and CCC haplotypes were no longer significant (β = −0.026, p = 0.575 and β = +0.011, p = 0.302, respectively) while the effect of the CTC haplotype was barely modified (β = +0.051, p = 2.01 10^−7^). The CCT and CCC haplotypes are the only two haplotypes carrying the rs13180-C allele, suggesting that these haplotypes were reflecting an effect of rs13180. This is in accordance with the nearly complete association between rs13180 and the best *cis* eSNP rs12592111 (r^2^ = 0.96).

For 60 probes (group C in Table S1), the best *cis* eSNP was not among the associated htSNPs and adjusting for its effect attenuated the haplotype association. For 10 probes, further adjustment on the second imputed best *cis* eSNP completely explained the haplotype association originally detected. This did not hold for the other 50 probes, suggesting that the original haplotypic signal had actually captured the effect of the originally untyped best *cis* eSNP, but this latter was not sufficient to characterize the full association observed at the probe locus. An example is given in Figure 4 with the *IREB2* gene. Three common haplotypes were associated with increased *IREB2* expression. After adjustment for the best imputed *cis* eSNP (rs12592111), two of them were no longer associated to expression as a consequence of the strong LD of one of the htSNPs (rs13180) with the best *cis* eSNP, while the third haplotype remained significant.

**Figure 5:**
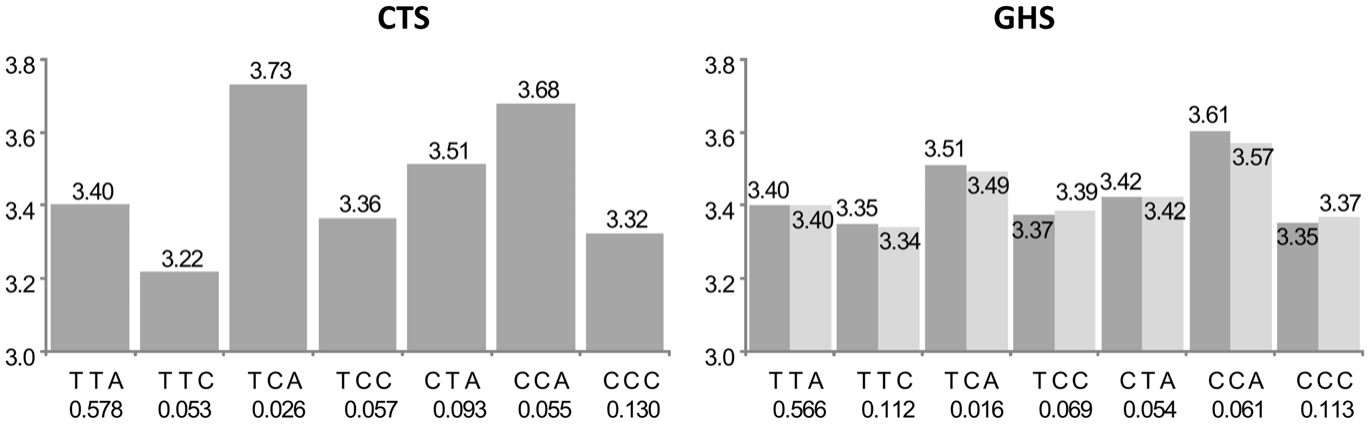
Association between ILMN_1689088 (*COLEC12*) expression and the main haplotypes derived from rs9966524, rs9960856, and rs2846666. See the first paragraph of legend in Figure 2 for explanations. In CTS, the rs9966524 and rs2846666 were substituted by their corresponding proxies, rs3932728 (r^2^ = 0.82) and rs2846667 (r^2^ = 0.87). The original haplotypic association (dark grey bars) was due to two haplotypes associated with increased *COLEC12* expression, the TCA (β = +0.331 p = 3.07 10^−10^ and β = +0.111, p = 5.83 10^−4^, in CTS and GHS, respectively) and the CCA (β = +0.278 p = 5.96 10^−18^ and β = +0.204, p = 4.19 10^−60^, in CTS and GHS, resp.). In GHS, the best imputed *cis* eSNP was rs11081136 whose minor allele was associated with increased *COLEC12* expression (β = +0.091, p = 1.02 10^−26^). After adjustment for rs11081136 (light grey bars), the TCA (β = +0.092, p = 2.52 10^−3^) and CCA (β = +0.171, p = 1.39 10^−40^) haplotypes were still associated with *COLEC12* expression. The rs11081136 effect also remained significant (β = +0.061, p = 1.12 10^−13^).

For the remaining 21 probes (group D in Table S1), the best *cis* eSNP was not among the associated htSNPs, and both the haplotypic and the best *cis* eSNP effects remained significant in the conditional model. Such patterns suggest that the imputation analysis revealed an additional independent signal that was not captured by the haplotype analysis on typed SNPs. An example is given in Figure 5 with the *COLEC12* gene. Two haplotypes were significantly associated with increased *COLEC12* expression, and these effects were barely modified by adjustment on the best *cis* eSNP rs11081136 whose effect also persisted in the conditional analysis.

In a final step, we tested whether the haplotype effects were compatible with additive effects of multiple SNPs or whether a more complex pattern could explain the observed haplotypic association. In the vast majority of cases (75 out of 105, 71%) the hypothesis of additive effects of multiple SNPs was not rejected (additive effects of 2 SNPs in 35 cases, 3 SNPs in 28 cases, and 4 SNPs in 12 cases). These situations were generally characterized by the presence of one SNP with a predominant effect on probe expression and additional SNPs with more modest effects. The remaining 30 haplotypic associations (29%) were not compatible with additive effects of typed or imputed SNPs.

For the discovery phase of this study, we had filtered out probes harboring common SNPs in their genomic sequence to avoid spurious associations due to differential binding of the probe to its target sequence. This filtering had been performed using the HapMap 2 SNP database as reference. Since imputation analyses were later conducted using the more recent 1000 Genome reference database which contains rarer SNPs, we checked whether the identified haplotypic associations might not be due to newly reported polymorphisms in the genomic sequence of the 105 probes with multiple *cis* eSNP effects. In 38 probes, the sequence was still devoid of SNPs. Four probes (ILMN_1683305, ILMN_1722698, ILMN_1741371, ILMN_2285618) were found to harbor an SNP with minor allele frequency (MAF) between 0.02 and 0.06, and for two of them (ILMN_1688305 and ILMN_1741371) this SNP was correctly imputed but not associated with probe expression (p = 0.01 and p = 0.55). For the remaining 63 probes, rare SNPs (MAF<5‰) were identified in the genomic sequences. However, such rare SNPs are unlikely to explain the multiple *cis* eSNPs associations observed in our study. Indeed, to explain at least 6% of probe variability, the minimum value observed for the 105 probes (Table S1), a SNP with a MAF<1% would have to be associated with an extremely strong genetic effect that would be characterized by outliers values in the probe expression distribution. We did not observe such outliers for any of the probes (Figure S2).

### Relevance to human diseases

Among the loci characterized by multiple *cis* htSNPs associated to probe expression, 24 were reported to be associated with human diseases or quantitative traits in the GWAS catalog [23] (Table S2). Notably, for 4 of these loci, the GWAS hit was among, or in complete LD (r^2^ = 1) with one of the identified *cis* htSNPs: *C1orf85* (locus for mean corpuscular hemoglobin concentrations [24]), *IREB2* (locus for chronic obstructive pulmonary disease [25]), *OPTN* (locus for Paget's disease) [26]) and *TSEN2* (locus for prostate cancer [27]).

A locus of particular relevance is *AP3S2* previously reported in a GWAS of type 2 diabetes [28] and associated in our study with *cis* haplotypes involving the additive effects of 3 SNPs, rs7173483, rs3803536 and rs1269077 (Figure 3 and Table S1). The lead SNP reported in the GWAS, rs2028299, was not among the identified *cis* htSNPs, although it was associated with *AP3S2* expression by single SNP analysis (p = 5.17 10^−7^ in CTS and p = 4.10 10^−17^ in GHS). However, when adjusting for the effects of the 3 htSNPs, the rs2028299 was no longer associated with *AP3S*2 expression (p = 0.986 and p = 0.289, respectively). The *cis* effect of rs2028299 was actually due to its LD with two haplotypes associated with increased *AP3S2* expression levels.

## Discussion

It is widely accepted that haplotype analysis built on several SNPs at a given locus presents several strengths: it can identify independent additive SNP effects, distinguish true effects from those due to LD between SNPs, suggest functional interaction between SNPs and identify the effect of untyped SNPs that are tagged by haplotypes. In order to better characterize the genetics of monocyte gene expression, we conducted the first genome-wide search for *cis* haplotype effects with comprehensive replication in an independent sample. This analysis was performed using two of the largest gene expression datasets available so far, the CTS and GHS resources. The search for *cis* haplotype effects was conducted using a statistical approach whose efficiency has already been demonstrated in the context of GWAS [19]. This methodology has the advantage of identifying both independent additive effects of *cis* eSNPs and more complex haplotype effects, whereas only the former can be identified through conditional GWES as recently proposed [11].

**Figure 6:**
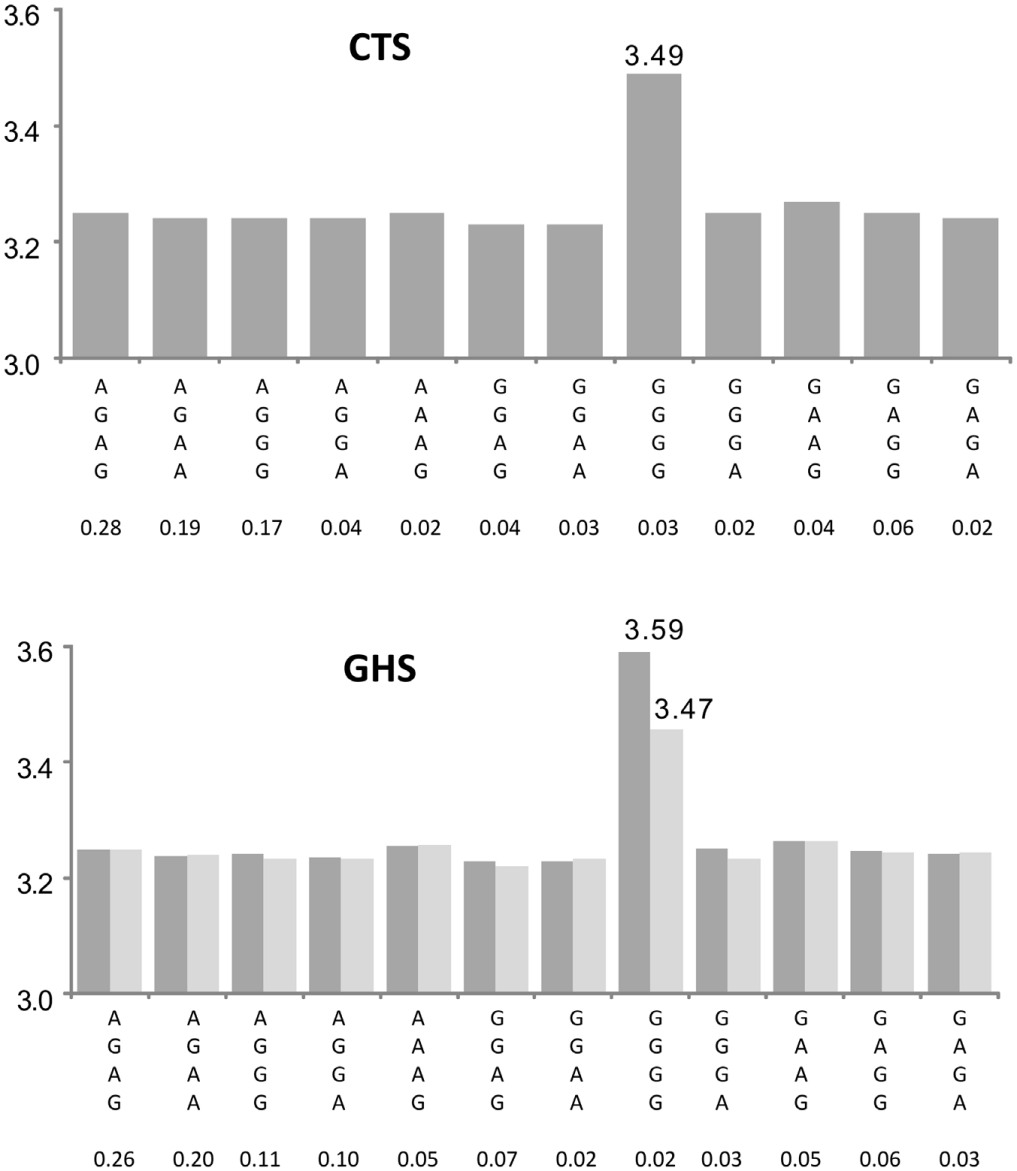
Association between ILMN_1757379 (*OPN1SW*) expression and the haplotypes derived from rs1109552, rs4731507, rs4731513, and rs339088. The top panel shows the results in the discovery cohort CTS and the bottom panel in the replication cohort GHS. Each bar corresponds to the expected mean of gene expression associated with one dose of the corresponding haplotype under the assumption of additive haplotype effects. According to this model, the expression level of an individual is the sum of the levels of his (her) two haplotypes. Haplotype frequencies are indicated under each haplotype label. In CTS, the rs4731507 and rs339088 were substituted by their perfect proxies (r^2^ = 1) rs4283986 and rs339085, respectively. The original haplotypic association (dark grey bars) was due to a unique rare haplotype derived from 4 common htSNPs. This rare haplotype, GGGG, was associated with a strong increase in *OPN1SW* expression (β = +0.240, p = 8.12 10^−26^ and β = +0.341, p<10^−307^ in CTS and GHS, respectively). After adjusting in GHS for the best imputed *cis* eSNP rs142976957 (light grey bars), the effect of this rare haplotype was still highly significant (β = +0.208, p = 4.78 10^−135^).

A key aspect of this work is that we did not apply in our discovery phase any correction for multiple testing. Rather, we focused on gene expression where the magnitude of the haplotypic association was much larger than that of the single SNP association and replicated the findings in an independent sample where Bonferroni correction was then applied to the subset of probes selected by the discovery phase. Note that this strategy led to the selection of some haplotypic associations that would not have passed a strict Bonferroni correction at the discovery phase (i.e. a p-value lower than 2.38 10^−11^). This is the case for example of *OPN1SW* (Table S1, Figure 6) where the haplotypic p-value was 3.47 10^−8^ but largely exceeded the p-value of the best *cis* eSNP (p = 0.0275). Using this 2-step approach, 47% of the haplotypic associations detected in CTS that could be tested for replication in GHS turned out to be significant. This high rate of replication can be explained by the fact only haplotypic associations having a much greater likelihood than single SNP associations were selected, but also by the greater power of the GHS study due to its larger sample size.

Among the 412 probes tested for replication in GHS, 105 (i.e ∼25%) met the Bonferroni-corrected threshold for considering that an eQTL is under the influence of multiple *cis* eSNPs. About three-quarters of these haplotype associations were shown to be the consequence of independent additive effects of several eSNPs. The last quarter might tag functional combinations of SNPs or rare variants not yet identified in the latest version of the 1000Genomes reference (eg Figure 6). The identified haplotypes do not necessarily imply functionality of the involved htSNPs. Indeed, these htSNPs were initially selected with respect to their tagging property for characterizing the genetic architecture of the mapped loci and not with respect to any possible causal role in gene function. Most of the identified htSNPs involved in *cis* effects could be replaced by other proxy SNPs (r^2^>0.90) as illustrated in Table S3. Only experimental works would answer the question of which of these SNPs are functional.

Twenty-four of the replicated probes (∼23%) mapped to loci previously reported to be associated with human traits through GWAS. Although this might reflect a shared biological mechanism in some cases, no general conclusion can be drawn from the observation of a co-localization between a GWAS hit and *cis* eSNPs contributing to gene expression. A GWAS hit can be one of the eSNPs contributing to gene expression, but not necessarily the one with the strongest *cis* effect. A GWAs hit can also be “artificially” found associated with gene expression just because of its LD with the *cis* eSNPs. Recent works have shown that GWAS-associated loci were enriched in *cis* eSNPs [29], [30] but such an enrichment appears largely dependent on the gene density in the region which favors coincidental associations (personal data). Whatever the causes underlying this enrichment, our study emphasizes the need, before embarking into functional validation experiments, to conduct in-depth haplotype analyses of entire GWAS-associated loci to get a more precise picture of the genetic regulation of gene expression and its possible link with human traits.

Several limitations of this work must be acknowledged. First, for computational reasons, our search for haplotype effects was limited to haplotypic combinations of up to 4 (not necessarily adjacent) SNPs. As shown in Table S1, the expression of 10 probes was found influenced by the contribution of 4 htSNPs plus the best imputed *cis* eSNP. This suggests that longer haplotypes may exert some *cis* effects. Second, our strategy was to select for replication probes where the haplotypic p-value was 10^4^ smaller than the single SNP p-value. This stringent threshold was applied to identify probes where haplotypes were likely to be more relevant than single SNP alone for explaining probe expression variability. In setting any threshold for a study one balances the risk of missing genuine loci versus taking a large number of potentially false loci forward into replication. Although we cannot exclude that we missed some true positives, the strategy we applied enabled us to detect and replicate probes where the added contribution of multiple *cis* effects to single SNP was as low as 1% of the probe expression variability (Table S1). Third, as all investigations were conducted under the assumption of additive effects of haplotypes on expression, haplotypes with dominant or recessive effects may have been missed. Fourth, only genotyped SNPs were used in the discovery stage as CTS had not been imputed at the time of this study. The use of imputed SNPs that could get single association p-values smaller than those of the typed SNPs would have led to less haplotypic signals passing our 10^4^ discovery threshold. Fifth, expression data from the discovery CTS cohort were adjusted for center but no other method controlling for population stratification was applied. However, because the replication study used imputed genotypes and controlled for population stratification, the risk of false positives due to the two last causes is greatly reduced. Conversely, if a lower proportion of false positives had passed the discovery phase, the number of probes selected for replication would have been smaller, resulting in a less stringent Bonferroni threshold and a greater power of the replication study. We cannot then exclude that other probes with multiple *cis* SNPs influence may have been missed. Lastly, because different genotyping arrays were used in the discovery and replication studies, less than 16% (i.e. 412/2,650) of the effects detected in CTS could be assessed for replication in GHS. Some of these associations that could not be further explored in GHS (∼17% = 387/2,238) map to disease-associated loci (Table S4) and their replication may warrant further efforts. Nevertheless, it is worth reminding that this work was not aimed at cataloguing all functional cis eSNPs influencing monocyte expression but rather at serving as a proof-of-principle demonstrating that gene expression variability could be influenced by the presence of more than one *cis*-acting SNP.

In conclusion, a comprehensive exploration of *cis* haplotype effects in monocytes eQTLs demonstrated that the expression of a substantial proportion of genes is associated with several *cis* eSNPs exerting either additive or more complex interactive effects. Among the genes whose expression was influenced by multiple SNPs, several were reported to be disease-associated loci by GWAS. This work further highlights the need for conducting both haplotype-based and 1000Genomes imputation-based *cis* eQTL analyses before elaborating functional studies at disease-associated loci. It would also be worthwhile to assess whether the multiple *cis* eSNPs identified at disease-associated loci could also associate with the disease and could then contribute to the missing heritability raised by the recent waves of GWAS [31], [32].

## Materials and Methods

This work was based on two large genome-wide expression and genotype datasets from the Cardiogenics Transcriptomic Study and the Gutenberg Health Study, the latter serving as a replication cohort for the former. For the present work the CTS dataset extensively described in [14], [33], [34] included 363 patients with coronary artery disease or myocardial infarction and 395 healthy individuals. The GHS investigation [9], [34]–[36] was conducted in a population-based sample of 1,374 healthy individuals.

### Ethics statement

All individuals were of European descent. They all gave written informed consent. Ethical approval was given by the local ethics committee and by the local and federal data safety commissioners.

### The Cardiogenics Transcriptomic Study

#### Genome-wide expression study

Monocyte were isolated from whole blood using CD14 micro beads (Miltenyi) and lysed in Trizol. RNA was extracted in chloroform and ethanol, washed in RNeasy columns and incubated with DNase I before extracted in RNase-free water. RNA was quantified by the Nanodrop method. Expression profiling was performed using the Illumina HumanRef-8 v3 beadchip array (Illumina Inc., San Diego, CA) containing 24,516 probes corresponding to 18,311 distinct genes and 21,793 Ref Seq annotated transcripts. mRNA was amplified and labelled using the Illumina Total Prep RNA Amplification Kit (Ambion, Inc., Austin, TX). After hybridization, array images were scanned using the Illumina BeadArray Reader and probe intensities were extracted using the Gene expression module (version 3.3.8) of the Illumina BeadStudio software (version 3.1.30). Raw intensities were processed in R statistical environment using the Lumi [37] and beadarray packages. All array outliers were excluded and only arrays with high concordance in terms of gene expression measures (pairwise Spearman correlation coefficients within each cell type >0.85) were included in the analyses.

#### Genome-wide genotype study

EDTA anticoagulated venous blood samples were collected from all participants. Genomic DNA was extracted from peripheral blood monocytes by standard procedures (Qiagen). Genome-wide genotyping was carried out using two Illumina arrays, the Sentrix Human Custom 1.2M array and the Human 610 Quad Custom array. SNP analysis was restricted to autosomal SNPs with minor allele frequency >0.01, call rate >0.95 and Hardy-Weinberg equilibrium testing p-value>10^−5^. After quality control, 522,603 SNPs were used for association analyses with expression.

In order to avoid spurious associations due to hybridization difference, the genome-wide analysis of *cis* haplotype effects was restricted to autosomal probes that were identified by the ReMOAT program [37] as not harboring SNPs (accordingto HapMap 2 SNP database) in their genomic sequence ( n = 19,805 autosomal probes).

### The Gutenberg Health Study (GHS)

#### Genome-wide expression study

Monocytic RNA was isolated from peripheral blood monocytes by negative selection using RosetteSep Monocyte Enrichment Cocktail (StemCell Technologies, Vancouver, Canada), Trizol extraction and purification by silica-based columns. Expression profiling was performed using the Illumina HT-12 v3 BeadChip (Illumina, CA, USA) and generated data were pre-processed using Beadstudio. The Lumi R package [38] was also used for processing expression data.

All probes identified in CTS were available for replication in GHS.

#### Genome-wide genotype study

For each participant, genomic DNA was extracted from buffycoats prepared from EDTA blood samples. Individuals were typed for genome-wide genotype data using the Affymetrix Genome-Wide Human SNP Array 6.0 (Affymetrix, CA, USA). SNPs with minor allele frequency <0.01, call rate <0.98 and Hardy-Weinberg equilibrium testing p-value<10^−4^ were excluded from the analysis. 675,350 quality-control checked SNPs were available for analysis in GHS.

### Statistical analysis

#### Discovery phase

In CTS, each probe was assessed for locus-specific haplotype effects using a multi-step approach adapted from a more general genome-wide strategy previously applied to coronary artery disease [19] and Alzeihmer disease [39]:

the first step consisted in identifying genotyped SNPs mapping within a 200 kb interval from the start and the end of the probe sequence and eliminating part of the redundancy due to LD by identifying a subset of haplotype tagging SNPs (htSNPs) mapping the probe sequence. For this purpose, a sliding-windows approach was adopted. Within each bin of ten adjacent SNPs, we characterized the haplotypic structure defined by common haplotypes (ie with frequency >0.02) and, from this, we selected a subset of SNPs sufficient to characterize more than 95% of the inferred haplotypes. Haplotype inference was performed by use of the Stochastic-EM algorithm developed for haplotype-based association analysis [40], [41]. This procedure was applied to all consecutive bins of ten SNPs overlapping the probe locus and led to the final selection of a set of htSNPs per probe.
for each probe and its associated set of htSNPs of maximum size 187 (see Figure S1), all haplotypic models derived from the combination of 1 to 4 htSNPs, not necessarily adjacent, were tested for association with probe expression. The model that minimizes the scaled Akaike Information Criterion (AIC) [40], [42] was selected as the most informative and parsimonious (“best”) model. The scaled AIC of a haplotypic model was defined as (−2 * log(likelihood(model))+2*k*) where *k* is the number of estimated haplotype effects. The likelihood ratio test statistic was then used to assess the significance of this “best” haplotypic model and the corresponding haplotypic p-value was assigned to the probe.
for each probe, this haplotypic p-value was then compared to the smallest p-value derived from all single SNP (not only htSNP) association analysis. Probes with haplotypic p-value 10^4^ smaller than the smallest single SNP association p-value were selected for replication in GHS. To increase the sensitivity of the haplotype analysis that can be penalized by the number of degrees of freedom of the haplotype tests, no threshold was applied to the value of the haplotypic p-value *per s*e. In all these analyses that were adjusted for the disease status, a linear model was used to investigate the additive effects of alleles or haplotypes on probe expression. Haplotype analyses were conducted using the GridHaplo software [19] implementing the aforementioned Stochastic-EM algorithm and available at http://genecanvas.ecgene.net. Single SNP association analyses were performed using R environment.

#### Replication phase

Each probe selected from the discovery phase in CTS as well as the htSNPs defining the best associated haplotypic configuration were checked for availability in GHS. If a given htSNP was not genotyped in GHS, we sought whether we could find a genotyped proxy SNP (r^2^>0.80) using the SNAP software [18]. Replication in GHS of the haplotypic signals observed in CTS was assessed using the THESIAS software [21] implementing the same statistical haplotype model as in the GridHaplo program. THESIAS was used to further check whether the haplotype effects were consistent (i.e. the same haplotypes associated with the same direction of effects on probe expression) across the two studied samples. Probes with haplotypic p-value significant after Bonferroni correction and consistent effects were considered as replicated.

To assess whether the replicated haplotype effects could reflect the effects of single SNPs either untyped or typed in GHS but not in CTS, further imputation analyses were conducted in GHS. Imputation of 15,865,541 bi-allelic polymorphic SNPs was conducted by the minimac software (release 2012-03-14) using the 1000G Phase I Integrated Release Version 2 Haplotypes reference panel. Of these imputed SNPs, 10,210,859 SNPs were inferred with good imputation quality (r^2^>0.3). SNPs located within 200 kb distance from a replicated probe sequence were then tested for association with its corresponding probe expression using a linear regression analysis in which allele dosage (continuous from 0 to 2 copies of the minor allele) of imputed SNPs was used as implemented in the mach2qtl software [22] (http://www.sph.umich.edu/csg/abecasis/MACH/download/). Conditional analyses were then conducted where haplotype effects were further adjusted for the SNP demonstrating the strongest single locus association p-value, ie. the best *cis* eSNP. Probes with conditional haplotypic p-value still significant after Bonferroni correction were considered to be under the genetic influence of multiple SNPs

In a final step, we tested whether the multiple *cis* eSNP effects were compatible with the additive effects of multiple SNPs by setting appropriate constraints on regression coefficients associated with haplotypes using nested likelihood ratio test statistics as implemented in THESIAS. In few instances, conditional haplotype analyses on the second best imputed *cis* eSNP were also performed.

Because negative selection was used to isolate monocytes in GHS, it cannot be ruled out that contamination by non-monocyte cells might influence gene expression variability. As a consequence, all statistical analyses performed in GHS were adjusted for surrogate variables controlling for cell purity as previously described [34]. Analyses were also adjusted for the first five principal components calculated from the GWAS dataset by use of the Eigenstrat program [43] in order to correct for uncontrolled population stratification.

## Supporting Information

Figure S1 Distribution of the number of SNPs per probe in the Cardiogenics Transcriptomics Study. In light grey is shown the distribution of the total number of SNPs within a 200 kb distance of any probe. In dark grey is shown the distribution of the corresponding htSNPs after discarding redundancy between SNPs due to strong linkage disequilibrium. The y-axis represents the number of probes harboring a given number of SNPs within a 200 kb distance (shown on the x-axis).
(TIF)

Figure S2 Box Plot representation of the expression variability at the 105 probes with multiple cis eSNPs effects in the Gutenberg Health Study.
(TIF)

Table S1 Gene expressions with statistical evidence of multiple *cis* eSNPs influence in the Cardiogenics Transcriptomics Study (discovery phase) that replicated in the Gutenberg Health Study. (1) Haplotypic Signal (HS) expressed as minus LOG of the P-value assessing the association between the best haplotypic model and probe expression. (2) Single Locus Signal (SLS) expressed as minus LOG of the smallest observed single SNP cis-association P-value. (3) Difference between (1) and (2) characterizing the gain of information brought by haplotypes compared to the “best” single SNP. (4) Single Locus Signal (SLS) expressed as minus LOG of the smallest observed single (genotyped or imputed) SNP cis-association P-value. (5) Percentage of probe expression variance explained by the “best” (genotyped or imputed) SNP in the Gutenberg Health Study. (6) Imputation quality criteria of the best SNP in the Gutenberg Health Study. (7) Haplotype Tagging SNPs characterizing the observed haplotypic associations. htSNPs that were found to be the best cis eSNPs are indicated in bold. (8) Haplotypic Signal (HS) expressed as minus LOG of the P-value. (9) Haplotypic Signal (HS) expressed as minus LOG of the P-value adjusted for the best cis eSNP. (10) Minimum set of SNPs (htSNPs and/or imputed SNPs) necessary to explain the observed multiple genetic effects. Best cis eSNPs identified in the single locus analysis are indicated in bold. Second best SNPs identified in a second round of conditional single locus association analyses are underlined. (11) Percentage of probe expression variance explained by the parsimonious model of several SNPs in the Gutenberg Health Study. (12) Difference between (10) and (5) characterizing the gain of information brought by multiple cis eSNPs compared to the “best” single cis eSNP. (13) Haplotype associations compatible with the independent and additive effects of two, three or four SNPs are shown as “A2”, “A3” and “A4”, respectively. Haplotype patterns that were not compatible with multiple independent additive (genotyped or imputed) SNPs are indicated as “NonA”. Probe results were separated into 4 groups: - Group A includes probes for which the best cis eSNP was among the identified htSNPs but was not sufficient to explain alone the observed haplotypic association. - Group B includes probes for which the htSNPs-derived mulitple genetic effects were still statistically significant and not modified after adjusting for the best cis eSNP which, conversely, was no longer significant. - Group C includes probes for which the htSNPs-derived multiple genetic effects were still statistically significant after adjusting for the best cis eSNP. However, the complexity of the multi SNPs model was reduced as the number of htSNPs required to characterize the association signal decreased when the best cis eSNP was included in the regression model. - Group D includes probes for which the htSNPs-derived multiple genetic effects were not modified by the adjustment on the best cis eSNP, the latter being also significantly associated with the probe expression.
(XLSX)

Table S2 List of probes with replicated multiple cis eSNP effects that map to disease-associated loci. Pairwise r^2^ were derived from the SNAP software database [18].
(XLSX)

Table S3 Known proxies for the identified htSNPs participating to cis haplotype effects. Proxies were identified by the SNAP software [18].
(XLSX)

Table S4 List of probes that could not be assessed for replication in GHS and that map to disease-associated loci.
(XLSX)
