# Supplementary figures and images for: Genome-Wide Haplotype Analysis of Cis Expression Quantitative Trait Loci in Monocytes
Source: PLoS Genet. 2013 Jan 31;9(1):e1003240. doi: 10.1371/journal.pgen.1003240 (PMC3561129; doi:10.1371/journal.pgen.1003240)

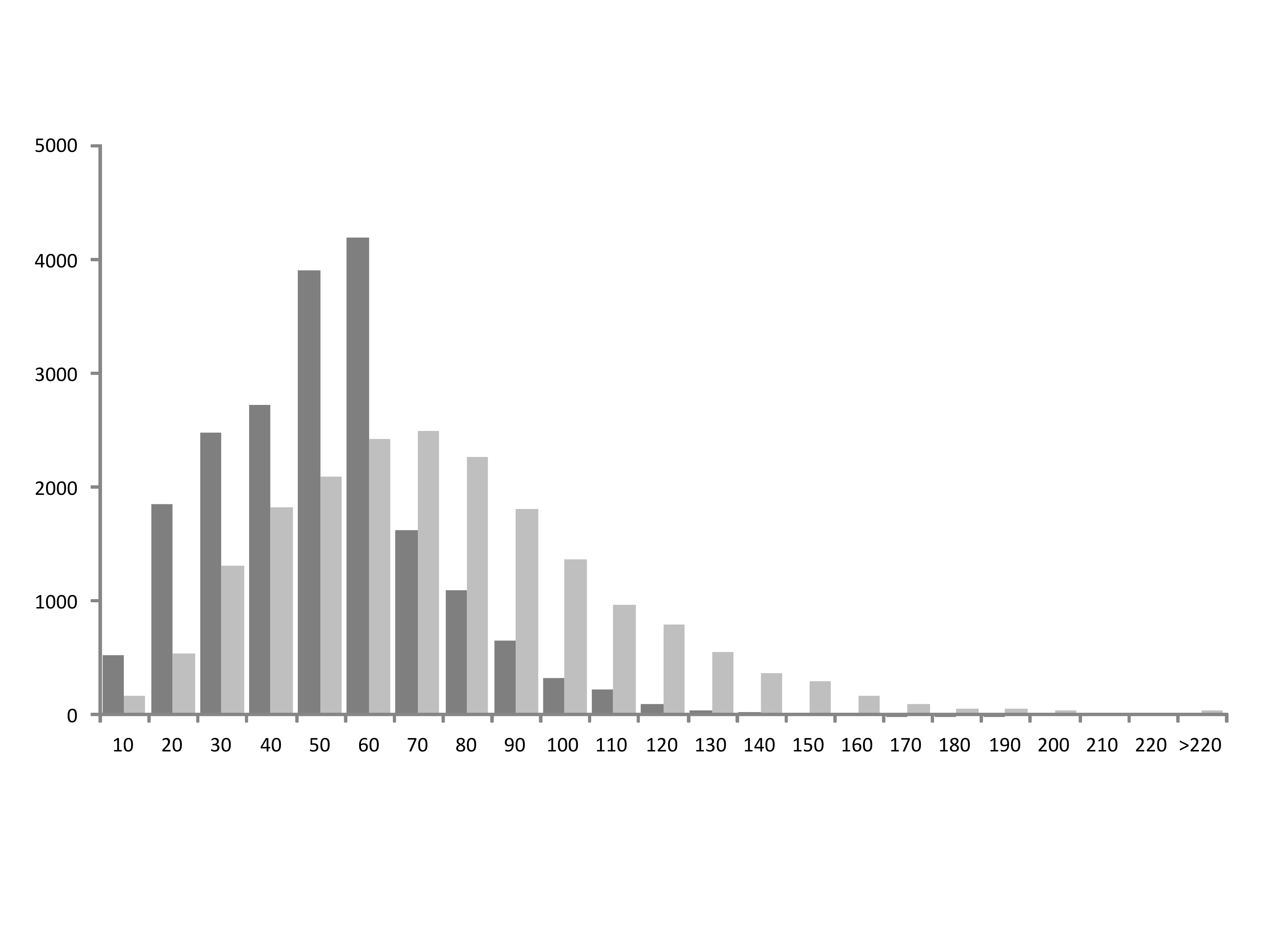

Supplement: Figure S1 — Distribution of the number of SNPs per probe in the Cardiogenics Transcriptomics Study. In light grey is shown the distribution of the total number of SNPs within a 200 kb distance of any probe. In dark grey is shown the distribution of the corresponding htSNPs after discarding redundancy between SNPs due to strong linkage disequilibrium. The y-axis represents the number of probes harboring a given number of SNPs within a 200 kb distance (shown on the x-axis). (TIF) [file pgen.1003240.s001.tif]

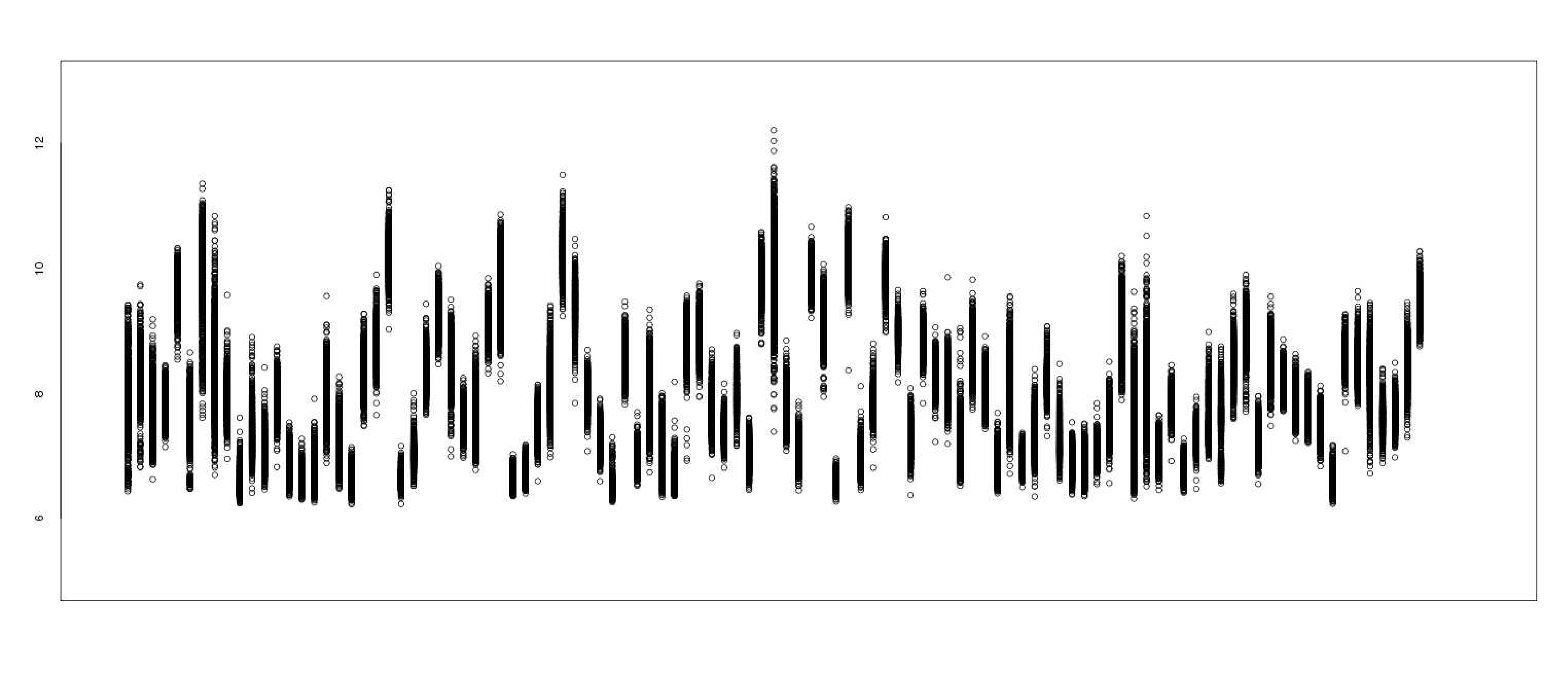

Supplement: Figure S2 — Box Plot representation of the expression variability at the 105 probes with multiple cis eSNPs effects in the Gutenberg Health Study. (TIF) [file pgen.1003240.s002.tif]
